# Editorial: Mechanisms by Which Acute and Chronic Exercise Promote Cardiometabolic Health

**DOI:** 10.3389/fcvm.2019.00159

**Published:** 2019-11-06

**Authors:** Jacob M. Haus, Bradford G. Hill

**Affiliations:** ^1^School of Kinesiology, University of Michigan, Ann Arbor, MI, United States; ^2^Division of Environmental Medicine, Department of Medicine, Diabetes and Obesity Center, Christina Lee Brown Envirome Institute, University of Louisville, Louisville, KY, United States

**Keywords:** exercise, physical activity, acute, chronic, metabolism, cardiovascular health and disease, cardiometabolic health

Exercise promotes cardiometabolic wellness and is effective in the prevention and treatment of cardiovascular disease, diabetes, and obesity-related conditions. Despite unequivocal evidence of the cardiovascular benefits conferred by exercise, the mechanisms by which the cardiovascular system adapts to exercise remain poorly understood. This Research Topic on *Mechanisms by which acute and chronic exercise promote cardiometabolic health* presents some of the latest ideas, arguments, and evidence from investigations at the molecular level to macroscopic observations on whole animals including humans.

We begin our compilation with a comprehensive review of the cardiovascular effects and benefits of exercise by Nystoriak and Bhatnagar which highlights the classic observations of decreased cardiovascular mortality and risk of developing cardiovascular disease in physically active individuals. These epidemiological observations are supported by robust physiology data on exercising individuals having lower blood pressure, higher insulin sensitivity, and a more favorable plasma lipoprotein profile—all of which are mechanistically supported by animal models of exercise. The authors continue with a “more is not always better” opinion where even though moderate levels of exercise have been found to be consistently associated with a reduction in cardiovascular disease risk, there is evidence to suggest that continuously high levels of exercise, such as marathon running, could have detrimental effects on cardiovascular health. Certainly, continued work is needed to identify the specific dose response relationship between the extent and duration of exercise and the reduction in cardiovascular disease risk and mortality.

The collection continues with a review by Pinckard et al. on how exercise enhances inter-organ communication through the release of myokines and their role in exercise-mediated adaptation. Myokines are chemical messengers that function in an autocrine, paracrine, or endocrine manner to influence crosstalk between different organs including skeletal muscle, liver, and adipose tissue. They are of great interest with regards to cardiovascular health because the well-known protective actions of exercise on cardiovascular function are at least partially mediated by increased secretion of myokines. The myokines reviewed include interleukin-6, myonectin, follistatin-like 1, and neuron-derived neurotrophic factor and how recent work has focused on potential therapies using exercise-induced myokines as a therapeutic strategy to improve cardiovascular function in patients who are unable or unwilling to exercise.

Certainly, there is ample evidence that regular exercise and increased physical activity are critical for health but are there limitations to exercise benefits in disease states such as peripheral artery disease? A compelling perspective is offered by Zakari et al. where the authors distinguish between the inherent capacity to perform, as compared to adaptive responses to active exercise training in relation to cardiovascular health and peripheral artery disease. The position of “exercise as medicine” has been advocated in the broad context as general medical care, but also in the specific context as a therapeutic, to be considered in much the same way as other drugs. As there are non-responders to many medications, there also are non-responders to exercise; individuals who participate but do not demonstrate appreciable improvement/benefit. In this review, the authors highlight the settings where the stress induced by exercise may aggravate an underlying condition, rather than attenuate chronic disease.

Although exercise promotes numerous salutary effects on the cardiovascular system, several human studies have concluded that the beneficial effects of regular exercise are attributable to a decrease in chronic inflammation and inflammatory mediator production. These clinical findings are complemented by animal studies that report regular moderate intensity exercise decreases the risk for chronic disease development through modification of the immune system. Given this backdrop, Chuong et al. ask the important question of whether or not changes in innate immunity underlie the cardiovascular benefits of exercise? Despite the immunoregulatory effects of exercise, the underlying cellular mechanisms and signaling pathways that promote cardiovascular health remain unknown. Identifying key determinants by which exercise modulates inflammatory responses will likely offer new therapeutic targets for the treatment of cardiovascular disease. Given that the innate immune system is responsible for initiating most inflammatory responses and causally contributes to cardiac pathology and repair, the opinion is presented that neutrophils and macrophages are critical in mediating the cardioprotective effects afforded by exercise.

While the periodic metabolic stress imposed by regular exercise appears fundamental in driving cardiovascular tissue adaptation, it remains to be fully elucidated as to how different types, intensities, or durations of exercise elicit different levels of metabolic stress and promote tissue remodeling. Fulghum and Hill discuss how exercise affects cardiac structure and function and how exercise-induced changes in metabolism regulate cardiac adaptation. They review the latest evidence on how exercise elicits an adaptive, beneficial form of cardiac remodeling that involves cardiomyocyte growth and proliferation. Conversely, the authors highlight how chronic levels of extreme exercise may increase the risk for pathological cardiac remodeling or sudden cardiac death. The mechanistic underpinnings of how acute and chronic cardiac adaptations to exercise involve the concept of metabolic periodicity, which appears important for regulating mitochondrial quality and function, for stimulating metabolism-mediated exercise gene programs and hypertrophic kinase activity, and for coordinating biosynthetic pathway activity.

Nuclear factor erythroid 2 like 2 (Nrf2) is the major stress response transcriptional regulator for antioxidant genes. Declined Nrf2-antioxidant signaling leads to accumulation of reactive oxygen/nitrogen species (ROS/RNS) and oxidative stress, which is either causally linked or associated with numerous health problems including diabetes and cardiovascular disease. Thus, targeting endogenous antioxidant defense mechanisms such as Nrf2 to enhance cytoprotection may be efficacious. Importantly, sustained physical activity or routine exercise training upregulates Nrf2-dependent cytoprotective targets in human skeletal muscle and mouse heart and provides a non-pharmacological strategy for combatting ROS/RNS at the cellular level. In this collection's only original research article, Shanmugam et al. tested the hypothesis that exercise-based activation of Nrf2, and its transcriptional regulation of antioxidants, protects the heart from isoproterenol-induced myocardial damage and dysfunction in mice. Isoproterenol is a beta-adrenergic receptor agonist and has been widely accepted as an inducer of pathologic cardiac remodeling in experimental animals by generation of excessive ROS, leading to alterations in cardiac metabolism and progressive myocardial injury. These data provide evidence for exercise-mediated enhancement of Nrf2 activity and the prevention oxidative stress mediated myocardial injury.

As mentioned above, Nrf2 is a transcriptional regulator for antioxidant genes. One such gene, GLO1 (glyoxalase 1), when downregulated, has been implicated in the pathology of complications associated with diabetes and perhaps a novel mechanism responsible for the development of insulin resistance. Mey and Haus continue the series with a mini review on the phenomenon of dicarbonyl stress and glyoxalase-1 downregulation and they ask what implications reduced GLO1 may have for insulin resistance, type 2 diabetes (T2D) and cardiovascular disease. The authors also emphasize the therapeutic potential for exercise intervention in restoring healthy GLO1 expression. Glyoxalase-1 (GLO1) is a ubiquitously expressed cytosolic protein which detoxifies reactive dicarbonyl metabolites and thus a consequence of reduced GLO1 protein expression is accumulation of carbonyl-modified proteins or dicarbonyl stress, which is elevated in obesity, insulin resistance and T2D. Recent work has identified the therapeutic potential of novel natural agents as a GLO1 inducer, which resulted in improved whole-body metabolism in obese adults. Given skeletal muscle is a major contributor to whole-body glucose, lipid, and protein metabolism, such GLO1 inducers may act, in part, through mechanisms in skeletal muscle. Current investigations examining the specificity of dicarbonyl stress and GLO1 biology in human skeletal muscle are lacking and this minireview summarizes the existing human literature examining skeletal muscle GLO1 and highlights the emerging therapeutic concepts for GLO1 gain-of-function in conditions such as insulin resistance and cardiometabolic disease.

It is not a new message that physical activity helps optimize blood glucose control, improve cardiovascular health, and reduce cardiovascular-related mortality, but increased awareness of the dangers of inactivity and refinement of physical activity advice is essential. While the benefits of increased physical activity are clear, what kind of exercise is best and when is the right time to move? To fully test our understanding of exercise dose on mitigating hyperglycemia, detailed studies of differential exercise time and intensity are needed. Solomon et al. conclude the series with a compelling argument that interrupting sitting time in the postprandial period is a desirable strategy to optimize blood glucose control and maximize cardiometabolic health. Experimental evidence demonstrates that interrupting inactivity (sitting time) with physical activity breaks is a successful approach for managing blood glucose levels but recommendations concerning the optimal time to interrupt sitting do not yet exist. Since postprandial hyperglycemia is an independent cardiovascular risk factor and since we spend many hours each day in a postprandial state, timing the interruption of sitting with physical activity to minimize postprandial fluctuations in blood glucose is a sensible approach to maximize cardiometabolic health. Approaches such as this will help curb the increasing incidence of diabetes and thereby improve cardiovascular health, longevity, and quality of life for the increasingly inactive global population.

It is our sincere hope that this Research Topic will not only provide readers with new insights and viewpoints on the issue of exercise and cardiometabolic health, but will also stimulate novel ideas, experiments, and further advances in this research field.

## Author Contributions

All authors listed have made a substantial, direct and intellectual contribution to the work, and approved it for publication.

### Conflict of Interest

The authors declare that the research was conducted in the absence of any commercial or financial relationships that could be construed as a potential conflict of interest.

